# Unconjugated Bilirubin exerts Pro-Apoptotic Effect on Platelets *via* p38-MAPK activation

**DOI:** 10.1038/srep15045

**Published:** 2015-10-13

**Authors:** Somanathapura K. NaveenKumar, Ram M. Thushara, Mahalingam S. Sundaram, Mahadevappa Hemshekhar, Manoj Paul, Chinnasamy Thirunavukkarasu, Basappa, Ganesh Nagaraju, Sathees C. Raghavan, Kesturu S. Girish, Kempaiah Kemparaju, Kanchugarakoppal S. Rangappa

**Affiliations:** 1 DOS in Biochemistry, University of Mysore, Manasagangothri, Mysuru-570 006, India; 2 Department of Internal Medicine, Manitoba Centre for Proteomics and Systems Biology, University of Manitoba, Winnipeg- R3E3P4, Canada; 3 Department of Biochemistry and Molecular Biology, Pondicherry Central University, Pondicherry-605 014, India; 4 Laboratory of Chemical Biology, Department of Chemistry, Bangalore University, Bengaluru-560 056, India; 5 Department of Biochemistry, Indian Institute of Science, Bengaluru-560 012, India; 6 Department of Studies and Research in Biochemistry, Tumkur University, Tumkur-572 103, India; 7 DOS in Chemistry, University of Mysore, Manasagangothri, Mysuru-570 006, India

## Abstract

Thrombocytopenia is one of the most frequently observed secondary complications in many pathological conditions including liver diseases, where hyperbilirubinemia is very common. The present study sought to find the cause of thrombocytopenia in unconjugated hyperbilirubinemic conditions. Unconjugated bilirubin (UCB), an end-product of heme catabolism, is known to have pro-oxidative and cytotoxic effects at high serum concentration. We investigated the molecular mechanism underlying the pro-apoptotic effect of UCB on human platelets *in vitro*, and followed it up with studies in phenylhydrazine-induced hyperbilirubinemic rat model and hyperbilirubinemic human subjects. UCB is indeed found to significantly induce platelet apoptotic events including elevated endogenous reactive oxygen species generation, mitochondrial membrane depolarization, increased intracellular calcium levels, cardiolipin peroxidation and phosphatidylserine externalization (*p* < 0.001) as evident by FACS analysis. The immunoblots show the elevated levels of cytosolic cytochrome c and caspase activation in UCB-treated platelets. Further, UCB is found to induce mitochondrial ROS generation leading to p38 activation, followed by downstream activation of p53, ultimately resulting in altered expression of Bcl-2 and Bax proteins as evident from immunoblotting. All these parameters conclude that elevated unconjugated bilirubin causes thrombocytopenia by stimulating platelet apoptosis via mitochondrial ROS-induced p38 and p53 activation.

Unconjugated hyperbilirubinemia is an incidental verdict on routine laboratory tests in most liver diseases including chronic liver disease, haemolytic jaundice, Gilbert’s syndrome and Crigler-Najjar syndrome^1^,^2^,^3^. Liver disorders quite often present haematological aberrations particularly thrombocytopenia (low platelet count). As high as 58% of liver disease patients are observed to have thrombocytopenia correlating increased concentration of bilirubin^4^. Consequently, the extent of thrombocytopenia is found to serve as an effective prognostic marker for liver diseases^4^,^5^. Thrombocytopenia not only leads to increased bleeding and haemorrhage, but also is an impediment to invasive procedures including diagnosis and therapeutic regimen. One of the reasons behind thrombocytopenia might be rapid destruction or clearance of platelets in the peripheral circulation. This is supported by various reports lately including our own, that demonstrate the intensely sensitive and fragile nature of platelets, as well as their tendency to undergo apoptosis in response to a wide array of intrinsic and extrinsic stimuli^6^. The anuclear platelets possess the necessary cellular machinery to undergo apoptosis like any other nucleated cell, with the exception of nuclear apoptotic events. The intrinsic apoptotic pathway is more commonly demonstrated in platelets, which transpires *via* mitochondrial dysfunction in response to stress and is presented with augmented levels of reactive oxygen species (ROS), mitochondrial membrane potential (ΔΨ*m*) depolarization, intracellular calcium ions (Ca^2+^), cytosolic cytochrome c (cyt. c), caspases −9 and −3 activities, and phosphatidylserine (PS) scrambling^7^,^8^.

Although the link between thrombocytopenia and liver diseases is quite apparent, at present the scientific community does not acknowledge liver disease as a probable cause of thrombocytopenia. Therefore, the present study sought to delve into the mechanism behind this phenomenon. One common hallmark in the liver diseases, including haemolytic jaundice, Gilbert’s syndrome and Crigler-Najjar syndrome, is elevated level of serum unconjugated bilirubin i.e. hyperbilirubinemia. Unconjugated bilirubin (UCB) is the reduced product of biliverdin and is an end-product of heme (Fe-protoporphyrin IX) catabolism. It is made in the Kupffer (hepatic) cells and in monocytic macrophages of bone marrow and spleen, which is then released into plasma^9^. UCB behaves as a physiological antioxidant in human extracellular fluids^10^,^11^. However, it is also demonstrated that bilirubin in the presence of the transition metal ion Cu (II) induces DNA strand cleavage via generation of ROS, particularly hydroxyl radical^12^. Hence, UCB acts as an antioxidant at normal physiological concentration and as a pro-oxidant at higher concentration, where it may reach up to 300 μM^11^. High concentration of UCB is linked to cell cytotoxicity and neurological dysfunctions^13^. On the other hand, there are studies that have reported the platelets undergo apoptosis in response to oxidative stress^14^,^15^,^16^. Accordingly, it is hypothesized that elevated levels of UCB during a disease condition might exert oxidative stress on platelets and thus trigger the process of apoptosis. Therefore, the present study demonstrates the pro-apoptotic potential of UCB on human platelets as an attempt to correlate hyperbilirubinemia and thrombocytopenia, and also explores the underlying molecular signalling pathway. We also report the observed incidences of thrombocytopenia in hyperbilirubinemia patients, as well as activated apoptotic markers in their platelets. Taken together, we demonstrate the clinical significance of UCB in depleting platelet count during hyperbilirubinemic conditions, which demands a collective treatment strategy considering the risks associated with thrombocytopenia during liver diseases.

## Results

### UCB induces oxidative stress and apoptosis in platelets

**Figure 1:**
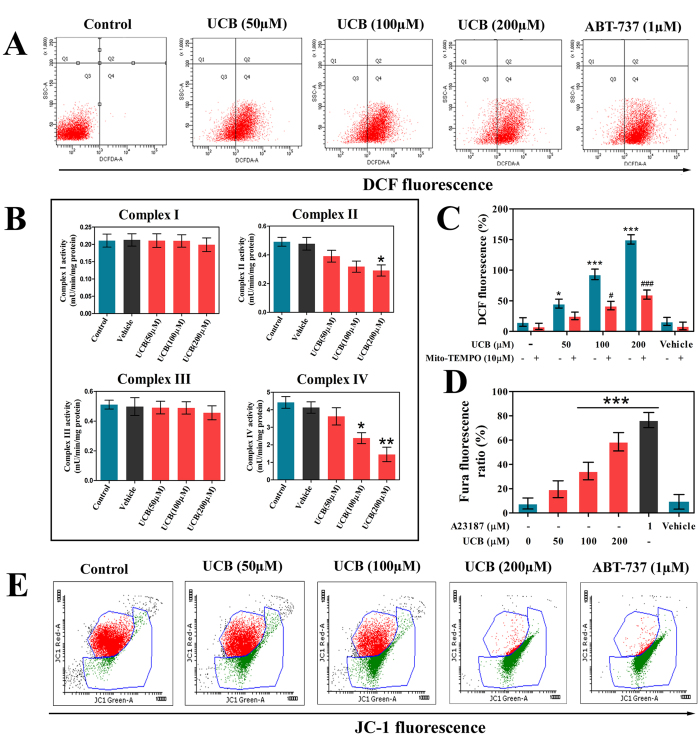
UCB induced oxidative stress and mitochondrial stress in human platelets. (**A**) Flow cytometric analysis of ROS in UCB treated platelets, (**B**) Effect of UCB on components of mitochondrial electron transport chain, (**C**) Effect of Mito-TEMPO on UCB induced ROS generation. (**D**) Flourometric analysis of intracellular calcium level and (**E**) Flow cytometric analysis of mitochondrial membrane potential. Values are presented as mean ± SEM (n = 5) and expressed as percentage increase in (**C**) DCF and (**D**) Fura fluorescence. */^#^ *p*< 0.05, **p < 0.01, ***/^###^ *p*< 0.001; *significant compared to control platelets; ^#^significant compared to UCB alone treated platelets.

**Figure 2:**
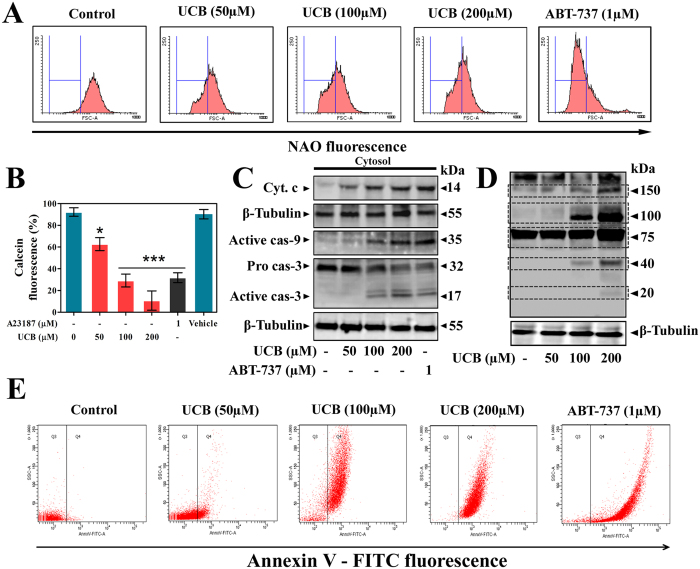
UCB induced mitochondrial dysfunction and apoptosis in human platelets. (**A**) Flow cytometric analysis of cardiolipin peroxidation in UCB treated platelets. (**B**) UCB induced mPTP formation (**C**) Immunoblots showing expression of cytochrome c, active caspase-9 and caspase-3. (**D**) Effect of UCB on protein phosphorylation. Membrane was cut based on the molecular weight, probed with antibody of interest and band of interest with molecular weight is indicated with an arrow. (**E**) Flow cytometric analysis of PS externalization in UCB treated platelets. Values are presented as mean ± SEM (n = 5), expressed as percentage decrease in (**B**) calcein. *p < 0.05, **p < 0.01, ***p < 0.001; significant compared to control platelets.

To investigate whether UCB affects platelet function and life span, we examined the oxidative stress and apoptotic markers of platelets in presence or absence of UCB. UCB-treated platelets were assessed for oxidative stress parameters like endogenous ROS generation, intracellular calcium content and ΔΨ*m* depolarization. FACS analysis of UCB-treated platelets showed concentration-dependent increase in ROS generation measured using DCF fluorescence (Fig. 1A). To rule out the interference of UCB fluorescence at the similar excitation and emission range of DCF, UCB -treated platelets were monitored for fluorescence without adding DCF dye. We found no observable fluorescence in UCB-treated platelets suggesting that UCB do not interfere with DCF fluorescence (Supplementary Figure 1). It is established that impaired functions of ETC complex is the primary event leading to mitochondrial ROS production^17^,^18^. Therefore, effect of UCB on ETC complex was evaluated. UCB significantly inhibited complex II and complex IV activities in a concentration-dependent manner (Fig. 1B). Additionally, ROS generation induced by disturbances in ETC was confirmed using Mito-TEMPO, a mitochondria targeted anti-oxidant. It significantly abolished UCB-induced endogenous ROS generation, indicating that mitochondria are the primary source of ROS in unconjugated hyperbilirubinemia (Fig. 1C). Fluorometric analysis of UCB-treated platelets showed concentration-dependent increase in intracellular calcium levels (Fig. 1D) together with decreased ΔΨ*m* (Fig. 1E). As discussed earlier, to exclude the interference of UCB with JC1 fluorescence, (used to detect ΔΨ*m*) unstained UCB-treated platelets were monitored for fluorescence and we observed no change suggesting UCB do not interfere with JC1 fluorescence (Supplementary Figure 2). Altered mitochondrial dysfunction and calcium homeostasis are marked with peroxidation of cardiolipin, release of cyt. c into cytosol via mPTP, downstream caspase activation and PS externalization. Accordingly, UCB triggered cardiolipin peroxidation (Fig. 2A) and mPTP formation as determined by calcein-CoCl_2_ quenching assay (Fig. 2B). Further, downstream apoptotic events like cyt. c release, activation of caspase-9 and caspase-3 were evaluated using immunoblotting (Fig. 2C). It is demonstrated that during apoptosis cellular proteins can be phosphorylated through the activation of protein kinases and then exposed to caspase cleavage^19^. Consistent with this notion, we observed increased phosphorylated protein content in UCB-treated platelets using immunoblot (Fig. 2D) and flow cytometric analysis endorsed that there was concentration-dependent PS externalization in UCB-treated platelets (Fig. 2E). In all the assays, vehicle control (DMSO) was found to be non-toxic to platelets and did not induce either apoptosis or oxidative stress to platelets.

**Figure 3:**
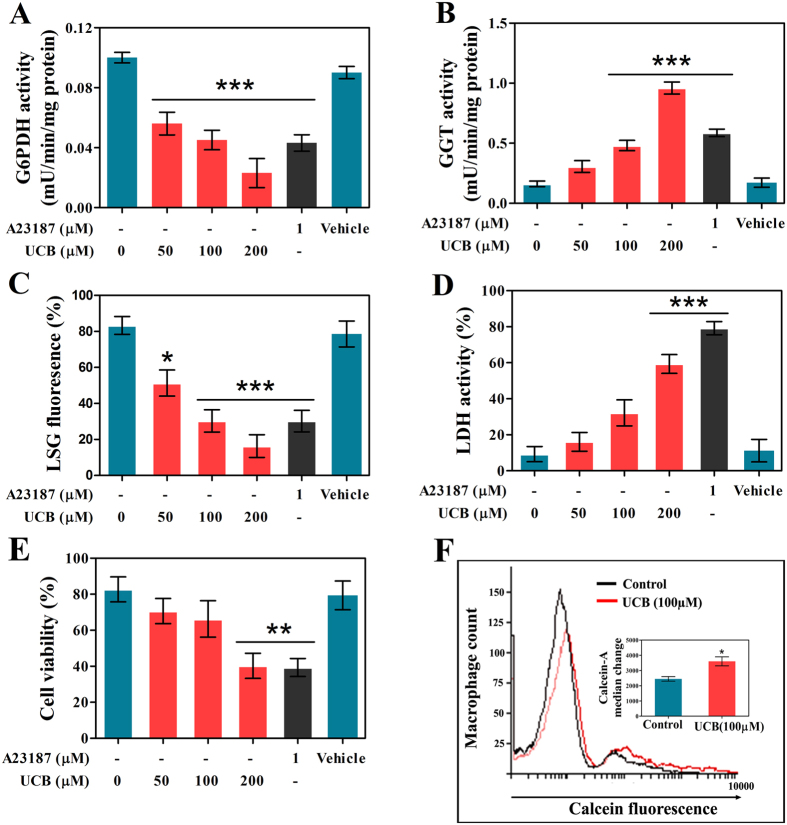
UCB induced cytotoxicity in human platelets. UCB induced oxidative stress and lysosomal alkalinisation as assessed by (**A**) G6PDH activity (**B**) GGT activity and (**C**) Lysosomal stability. Cytotoxic properties of UCB as determined by (**D**) LDH leakage (**E**) MTT cell viability assay and (**F**) Flow cytometric analysis of macrophages mediated engulfment of calcein-labeled control and UCB (100 μM) treated platelets. Values are presented as mean ± SEM (n = 5), expressed as percentage decrease in (**C**) LysoSensor Green DND-189 fluorescence. *p < 0.05, **p < 0.01, ***p < 0.001; significant compared to control platelets.

In order to assess the toxicity of UCB towards platelets, UCB-treated platelets were assessed for their viability. There was a significant decrease in G6PDH activity (Fig. 3A), increase in GGT activity of platelets (Fig. 3B) suggesting that platelets were in oxidative stress due to UCB treatment. The increased levels of lysosomal alkalinization, LDH release and MTT assay in platelets confirm the cytotoxic effect of UCB on platelets and a significant reduction in the viability of UCB-treated platelets (Fig. 3C–E). Further, we evaluated the macrophage-mediated clearance of UCB treated platelets by FACS analysis. We observed a significant increase in calcein fluorescence in macrophages incubated with UCB-treated platelets (Fig. 3F) suggesting that platelets undergo apoptosis upon UCB treatment.

Our next question was to determine whether UCB treatment activates platelets. To verify the activation of platelets, platelet aggregation and platelet adhesion assays were performed. UCB did not induce platelet aggregation by itself; however, it inhibited collagen-induced platelet aggregation in washed platelets at a lower dose (Supplementary Figure 3A–C). In addition, UCB altered the adhesive property of washed platelets towards collagen (Supplementary Figure 3D). Together, these findings attest a prior knowledge that UCB affects platelet function and primes them into apoptosis and not their activation.

### UCB-induces platelet apoptosis through p38-mediated mitochondrial dysfunction

**Figure 4:**
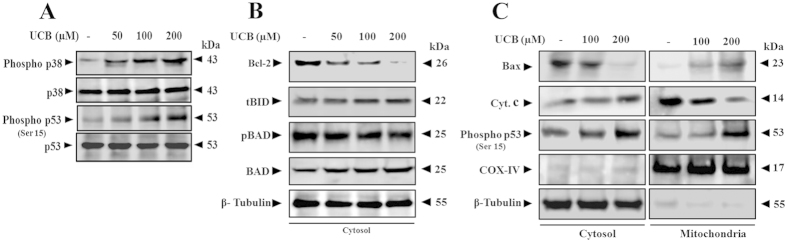
Differential expression of signalling proteins and mitochondrial accumulation of phosphorylated p53 in UCB treated platelets. (**A**) Immunoblots showing expression of phospho p38 and phospho p53 in UCB treated platelets. (**B**) Effect of UCB on cytosolic Bcl-2 family proteins expression (Bcl-2, tBid, phospho BAD and BAD) in platelets. (**C**) Expression of, Bax, cyt. C and phospho p53 in both mitochondria and cytosolic fractions of UCB treated platelets. COX-IV indicates cytochrome c oxidase subunit-IV used as mitochondrial loading control and β-tubulin as loading control for cytosolic fractions. Membrane was cut based on the molecular weight, probed with antibody of interest and band of interest with molecular weight is indicated with an arrow.

Apoptosis in UCB-treated platelets was so profound that nearly half of the treated population had undergone death as conferred by PS externalization and MTT assay. Therefore, we found it interesting to delineate the molecular mechanism of UCB-induced platelet apoptosis; hence phosphorylation of proteins involved in apoptosis signalling cascade was evaluated using immunoblotting. It is well established in platelets that MAP kinases play a significant role during mitochondrial dysfunction-mediated apoptosis^20^. Interestingly, there was increased phosphorylation of p38 (Thy180/Tyr182) as well as phosphorylation of p53 (Ser15) in platelets treated with UCB (Fig. 4A). Alongside, there was significant up-regulation of pro-apoptotic Bcl-2 family protein Bax, together with down-regulation of pro-survival proteins Bcl-2, phospho Bad, demonstrating that mitochondrial dysfunction was mediated by p53 (Fig. 4B). Further, there was an increased localization of Bax and phospho p53 in mitochondrial fraction and cyt. c in cytosolic fraction of platelets treated with UCB suggesting that p53 has a definite role in mitochondrial dysfunction (Fig. 4C).

**Figure 5:**
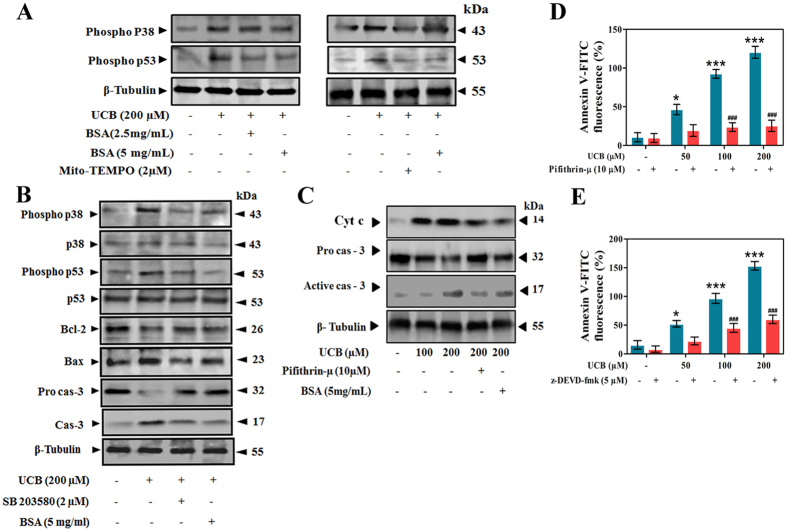
Inhibition of UCB mediated platelet apoptosis by different inhibitors. Immunoblots showing the effect of (**A**) BSA and Mito-TEMPO, a mitochondrial ROS quencher (**B**) SB203580 (specific p38 inhibitor) and (**C**) Pifithrin-μ (specific p53 inhibitor) on UCB induced platelet apoptosis. Effect of (**D**) Pifithrin-μ and **(E**) z-DEVD-fmk (specific caspase-3 inhibitor) on UCB induced PS externalization. Membrane was cut based on the molecular weight, probed with antibody of interest and band of interest with molecular weight is indicated with an arrow. *p < 0.05, ***/^###^ *p*< 0.001; *significant compared to control platelets; ^#^significant compared to UCB alone treated platelets.

In order to confirm the involvement of MAPK kinases in UCB-triggered platelet apoptosis, specific inhibitors of MAPK pathway were used. Under physiological conditions, albumin binds to UCB in circulation and hence inhibits its action towards the cells that are in close proximity. As expected, the immunoblotting data showed the decreased phosphorylation of p38 and p53 in UCB-BSA conjugate-treated platelets (Fig. 5A). Additionally, Mito-TEMPO (mitochondrial ROS scavenger) also inhibited p38 and p53 phosphorylation, indicating that their activation was due to mitochondrial ROS production (Fig. 5A). To explore whether p53 was being activated by p38, SB203580 a specific p38 inhibitor was used. Phosphorylation of p53 was inhibited by SB203580 suggesting that the phosphorylation was mediated by p38 activation (Fig. 5B). Further to confirm that UCB mediates apoptosis in platelets through p38 MAPK, the downstream apoptotic markers like Bcl-2, Bax and caspase-3 expression were evaluated both in presence and absence of SB203580 (Fig. 5B). Interestingly, SB203580 restored the altered levels of Bcl-2, Bax and caspase-3 in UCB-treated platelets as compared to untreated platelets. Furthermore, Pifithrin-μ, a specific blocker of p53- Bcl-xL/Bcl-2 interaction, inhibited cyt. c release and caspase-3 activation, and increased PS levels in UCB-treated platelets as compared to untreated platelets (Fig. 5C), indicating that UCB triggers apoptosis in platelets via p53-mediated activation of Bcl-2 family proteins and caspases. The specific caspase inhibitor z-DEVD-fmk, also restored the elevated PS levels establishing further that UCB- induced platelet apoptosis is caspase dependent (Fig. 5D,E).

### UCB alters platelet apoptosis markers in rat model

**Figure 6:**
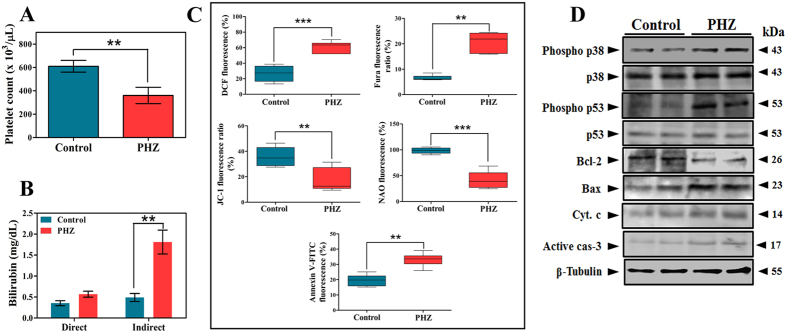
Phenylhydrazine induced hyperbilirubinemia in experimental animals. Figure showing decreased circulating platelets and increased platelet apoptotic markers during phenylhydrazine (PHZ) induced hyperbilirubinemia. Effect of hyperbilirubinemia on (**A**) Platelet count and (**B**) Serum direct and indirect bilirubin level in control and PHZ treated rats. (**C**) Elevated apoptotic markers in washed platelets obtained from control and PHZ treated rats as assessed by fluorometric assays and presented as percentage increase/decrease in fluorescence (ROS, intracellular Ca^2+^, ΔΨ*m*, cardiolipin peroxidation and PS externalization). (**D**) Expression levels of cytosolic apoptotic proteins in washed platelets of control and PHZ treated rats. Membrane was cut based on the molecular weight, probed with antibody of interest and band of interest with molecular weight is indicated with an arrow. **p < 0.01, ***p < 0.001; significant compared to saline control rats.

**Figure 7:**
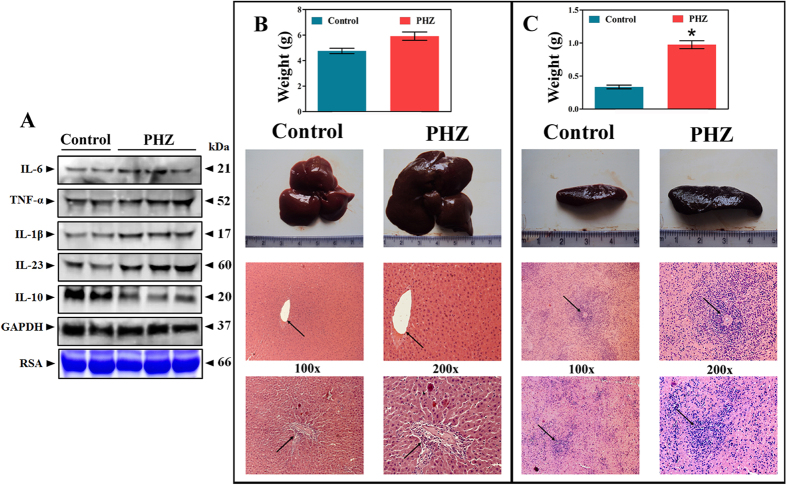
The serum cytokine levels, morphology and photomicrographs of liver and spleen tissues of control and PHZ induced hyperbilirubinemic experimental rats. (**A**) Altered serum cytokine levels in control and PHZ treated rats. Membrane was cut based on the molecular weight, probed with antibody of interest and band of interest with molecular weight is indicated with an arrow. GAPDH and rat serum albumin (RSA) were used as loading controls. The gel in this figure is cropped and the full length gel is presented in supplementary Figure 6. (**B**) Liver weight, morphology and photomicrographs of liver sections (H&E stained) of PHZ induced hyperbilirubinemic experimental rats. Arrow indicates damaged foci with large number of inflammatory cells in the sinusoid indicative of congestion as compared to saline control rats showing normal foci. (**C**) Spleen weight, morphology and photomicrographs of H&E stained spleen sections of PHZ induced hyperbilirubinemic experimental rats. Arrow indicates damaged spleenic nodule and germinal center of white pulp with constricted central artery as compared to saline control rats. *p < 0.05; significant compared to control rats.

Since, it was clear from the previous set of results that UCB induces platelet apoptosis, it was ideal to ascertain the effects of UCB *in vivo*. Therefore, phenylhydrazine (PHZ)-induced hyperbilirubinemic animals were initially evaluated for platelet count. PHZ administration effectively induces haemolytic hyperbilirubinemia in the rats, which is close enough to human haemolytic jaundice with elevated UCB levels^21^. We observed a significant decrease in the population of circulating platelets (Fig. 6A) with elevated serum UCB levels (Fig. 6B). The platelet apoptotic markers like endogenously generated ROS, intracellular calcium, ΔΨ*m*, cardiolipin peroxidation and PS externalization were also altered in PHZ-induced hyperbilirubinemic rats in a similar pattern as the *in vitro* results (Fig. 6C). In the previous set of experiments, activation of p38 and p53 were shown to be critical in UCB-mediated mitochondrial damage, hence platelets from the hyperbilirubinemic rats were evaluated for the same. Increased phosphorylation of p38 and p53 along with decreased Bcl-2 levels and increased levels of Bax, cyt. c and caspase-3 were clearly evident from respective immunoblots suggesting that the decreased platelet count was due to hyperbilirubinemia (Fig. 6D). To exclude the fact that PHZ itself interferes with altered platelet properties, platelets were treated with PHZ *in vitro*. As the results demonstrate, the alterations in ROS content, intracellular calcium levels, ΔΨ*m*, cardiolipin peroxidation, mPTP formation, PS externalization and LDH activity were found to be negligible (Supplementary Figure 4). In addition, altered serum cytokine levels were also observed in PHZ-induced hyperbilirubinemic rats compared to control (Fig. 7A). A significant difference in macroscopic and microscopic structure of both liver (Fig. 7B) and spleen (Fig. 7C) was observed in PHZ-induced hyperbilirubinemic rats compared to saline control.

### UCB-triggers platelet apoptosis in hyperbilirubinemic human subjects

**Figure 8:**
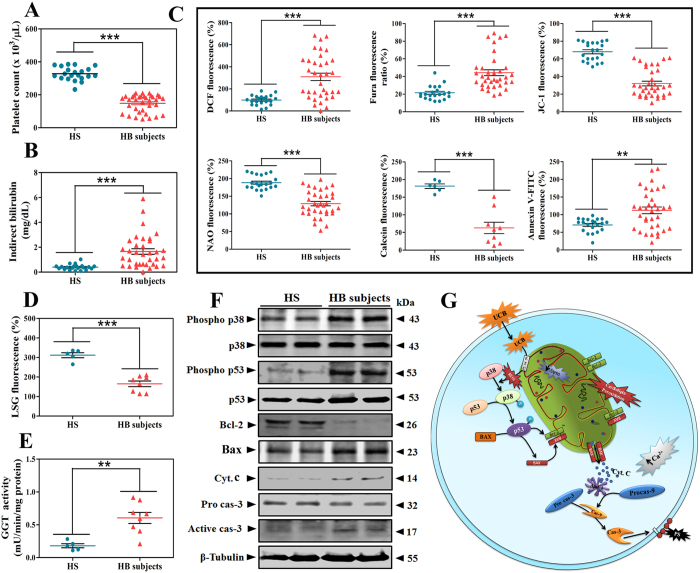
Effect of hyperbilirubinemia on platelets, platelet apoptotic markers, stress markers and expression of apoptotic proteins in human hyperbilirubinemic subjects. Effect of hyperbilirubinemia on (**A**) Platelet count and (**B**) Serum indirect bilirubin level in HS (n = 21) and HB (n = 35) subjects. (**C**) Hyperbilirubinemia induces platelet apoptosis in HB subjects as assessed by fluorometric assays and presented as percentage increase/decrease in fluorescence [ROS, intracellular Ca^2+^, mitochondrial membrane potential, cardiolipin peroxidation, mPTP formation in HS (n = 5) and HB (n = 8) subjects and PS externalization]. Effect of hyperbilirubinemia on (**D**) lysosomal stability as expressed as percent decrease in LysoSensor Green DND-189 fluorescence. (**E**) GGT activity in HS (n = 5) and HB (n = 8) subjects. (**F**) Cytosolic apoptotic protein expression levels in platelets of HS and HB subjects. β-tubulin was used as loading control. Membrane was cut based on the molecular weight, probed with antibody of interest and band of interest with molecular weight is indicated with an arrow. (**G**) Schematic representation of proposed molecular mechanism of UCB induced platelet apoptosis. Values are presented as mean ± SEM and expressed as percentage increase/decrease in DCF, JC-1, fura, NAO and calcein fluorescence. **p < 0.01, ***p < 0.001; significant compared to HS.

Further, to validate the link between thrombocytopenia and hyperbilirubinemia, hyperbilirubinemic (HB) patients were recruited and the correlation between indirect bilirubin level and platelet count was determined. As hypothesized, there was a significant decrease in the number of circulating platelets (Fig. 8A) along with elevated UCB levels (Fig. 8B) in HB subjects compared to HS. Therefore, platelet apoptotic markers and intracellular signalling events were evaluated in HS and HB subjects. There was significant alteration in platelet apoptotic markers like endogenous generation of ROS, intracellular calcium, ΔΨ*m*, cardiolipin peroxidation, mPTP formation and PS externalization (Fig. 8C). The obtained results were further backed up by demonstrating the decreased lysosomal acidity (Fig. 8D) and increased GGT activity (Fig. 8E). Moreover, the phosphorylated forms of p38 and p53 were also elevated significantly together with apoptotic markers like decreased Bcl-2 and increased levels of Bax, cyt. c and caspase-3 (Fig. 8F). The obtained results manifestly demonstrate the subliminal path of UCB priming circulating platelets into their deaths via an intricate cellular mechanism (Fig. 8G).

## Discussion

The current study for the first time, probed the cause of thrombocytopenia in unconjugated hyperbilirubinemia. Unconjugated hyperbilirubinemia is a common characteristic feature in liver diseases such as haemolytic jaundice, Gilbert’s syndrome and Crigler-Najjar syndrome^1^,^2^,^3^. Thereby, it is conjectured that there might be a link between hyperbilirubinemia and thrombocytopenia. Of note, thrombocytopenia is frequently observed in 58% of patients with liver diseases, which correlates with increased concentration of bilirubin^4^. The consequences of thrombocytopenia include increased risk of internal and external bleeding, delay in wound healing and coagulation defects. Moreover, it can become a clinically significant predicament in liver disease patients who have to undergo invasive diagnosis/therapy, interferon treatment, liver transplantation, cancer chemotherapy and surgery^22^. Though UCB is a metabolic waste product, it is also shown to be an endogenous antioxidant in mammalian tissues, and makes up a greater part of the antioxidant activity in human serum^10^,^11^. Bilirubin and biliverdin are potent modulators of cell signalling as well^23^,^24^. Nevertheless, unconjugated hyperbilirubinemia is associated with pathological conditions such as, increased haemolysis or haemolytic jaundice, Gilbert’s syndrome, and Crigler-Najjar syndrome^3^. UCB is also shown to exert cytotoxic and pro-apoptotic effects on neural cells and tumour cells^25^. Therefore, the present study investigated whether UCB affects platelet life span to trigger thrombocytopenia in liver diseases associated with elevated UCB levels. The results of the present study indicate that UCB induces platelet apoptosis *in vitro* at the concentration range 0–200 μM, due to the presence of UCB-binding proteins like albumin and other plasma proteins we deliberate our data with high concentration of UCB (0–200 μM). The mitochondrial/intrinsic pathway of apoptosis was mainly probed because of its susceptibility to physiological and pathological oxidative stress-inducing factors. Bilirubin is reported to be as efficient as anti-oxidants like glutathione and vitamin E in foraging hydroxyl radicals. However, elevated serum levels of UCB often exhibit pro-oxidative effect, which might be responsible for its anti-microbial properties. In the present study, UCB was found to considerably trigger the endogenous generation of ROS in platelets. Previously, few anti-oxidants such as resveratrol, sesamol and melatonin were also reported to trigger platelet apoptosis via ROS generation^6^. High level of UCB can lead to kernicterus (brain dysfunction) and studies suggest that oxidative stress is an important factor in bilirubin-induced neurotoxicity^26^. ROS plays a pivotal role in setting off the apoptotic events in platelets *via* the mitochondrial pathway by ΔΨ*m* dissipation. Mitochondria perform the function of biological switches that decide the fate of the respective cells by supplying the required energy for cell survival, and when there are lethal stimuli they trigger cell death^27^. The current results show that UCB significantly stimulates cardiolipin oxidation, ΔΨ*m* depolarization, which underscores the noxious effect of UCB on mitochondria. Bilirubin was previously reported to be toxic to astrocytes and neurons by causing damage to mitochondria and thus, leading to impaired energy metabolism and apoptosis^28^. Furthermore, oxidative stress can cause perturbations in intracellular Ca^2+^ homeostasis. At high concentrations (>1 μM), Ca^2+^ could inhibit respiration, cause cardiolipin oxidation, mitochondria to undergo permeability transition and release pro-apoptotic proteins leading to cell death^29^,^30^. Cardiolipin oxidation is a key event in mitochondrial dysfunction as well as in the early stages of the mitochondrial apoptotic pathway as it is the primary target of ROS^31^. Externalization of oxidized cardiolipin from the inner mitochondrial membrane to the outer, allows mPTP formation resulting in cyt. c leakage into the cytosol^32^. In the present study UCB was found to bring forth a remarkable increase in intracellular Ca^2+^ concentration and cardiolipin oxidation. Endoplasmic reticulum stress-induced perturbation in Ca^2+^ homeostasis and ROS generation leading to hepatocyte apoptosis has been observed as a pathological event in several liver diseases. The transcription factor C/EBP homologous protein, the mitogen activated protein kinase c-jun N-terminal kinase (JNK), Bcl-2 family proteins and caspase activation has been linked to ER-stress induced apoptosis^33^,^34^. UCB was found to decrease the activity of complex II and IV of ETC, increase mPTP formation and cytosolic cyt. c. Further, it was also observed to up-regulate pro-apoptotic Bcl-2 family protein Bax and tBid, down-regulate Bcl-2, activate caspases-9 and -3 and increase protein phosphorylation in a dose-dependent manner in platelets. Activated caspases can cleave a wide range of cellular substrates to expose new sites on proteins for phosphorylation^19^ and hence, increased levels of tyrosine-phosphorylated proteins are observed in UCB-treated platelets. Furthermore, UCB was capable of provoking PS externalization, an indispensable biochemical feature of an apoptotic cell that signals the phagocytic cells, finally leading to cell death. To rule out the possibility of exteriorized PS as a consequence of platelet activation, platelet aggregation studies were carried out wherein UCB was found to have no influence on platelet aggregation. UCB can easily enter the cells by simple passive diffusion and trigger toxicity^35^. Hence, overall toxic effects of UCB towards platelets were further confirmed by the positive results for MTT and LDH assays, decreased G6PDH activity and lysosomal stability, and increased GGT activity. G6PDH is involved in the generation of NADPH responsible for the recycling of glutathione and thus combating oxidative stress. Decreased platelet G6PDH activity in oxidative stress-induced pathologic conditions and in neonatal indirect hyperbilirubinemia has been reported^36^,^37^. Similarly, GGT is shown to cleave GSH into glutamic acid and cysteinylglycine, resulting in GSH depletion; hence, elevated levels of GGT indicate ongoing oxidative stress^38^. If the platelets are undergoing apoptosis, it should be cleared by the reticuloendothelial system through phagocytosis. Several studies have demonstrated the phagocytosis of apoptotic platelets using macrophage^39^,^40^,^41^. In the present study, phagocytosis of apoptotic platelets by macrophages was observed and confirms the UCB induced platelet apoptosis. Altogether, the findings suggest the platelet pro-apoptotic nature of UCB *in vitro*. The study was further extended to *in vivo* PHZ-induced hyperbilirubinemia rat model as well as hyperbilirubinemic human subjects. Interestingly, in both the cases it was found that there was not only a marked decrease in platelet count, but also all the above-mentioned events of mitochondrial apoptotic pathway were observed. These findings provide a substantial evidence for the pro-apoptotic effect of UCB *in vivo.*

The next phase of the study aimed to probe the molecular mechanism of UCB-induced platelet apoptosis. Various stress stimuli including ROS can activate JNK and p38 MAPK signalling. Platelets have also been shown to comprise of key signalling proteins (Bcl family proteins), which regulate mitochondria-mediated apoptosis^34^. Tang *et al.*^42^ have also reported the phosphorylation of p38 in human platelets by elevated glucose levels, which in turn phosphorylate p53. Both expression of p53 and its phosphorylation are shown both in mitochondrial and cytosolic platelet fractions^43^. In addition, it is demonstrated that activated p53 induces mitochondrial damage by interacting with Bcl-xL. It was studied using pifithrin-μ, a p53-specific inhibitor that inhibits p53 binding affinity towards mitochondrial Bcl-xL/ Bcl-2 as well as inhibits p53-mediated mitochondrial dysfunction and apoptosis^44^. On the other hand, it was previously reported that UCB induces oligodendrocyte precursor cell death via JNK activation, mitochondrial dysfunction and ROS generation^45^. In our study, UCB was found to trigger the phosphorylation of p38 and p53 in a dose-dependent manner. From the *in vitro* findings of the study, the molecular pathway was worked out as: UCB induces mitochondrial ROS generation, which in turn activates p38 and then causes the downstream activation of p53, eventually resulting in up-regulation of pro-apoptotic protein Bax and down-regulation of pro-survival proteins Bcl-2 and phospho Bad. Further, specific inhibitors of p38 and p53 evidently demonstrate that UCB induces apoptosis in platelets via p38 and p53 activation. Hence, we clearly demonstrate that activation of p53 is the ultimate key downstream event in UCB-driven apoptosis in platelets. In addition, we also reveal that mitochondria are the chief resources for ROS production in platelets and are accountable for UCB-induced p38 and p53 activation, and platelet toxicity by using Mito-TEMPO. The findings were further confirmed through *in vivo* rat model as well as hyperbilirubinemic human subjects.

The study also demonstrates that UCB induces apoptosis in platelets, but did not activate them as evident from platelet aggregation assay. UCB did not affect platelet aggregating properties, however it inhibited collagen-induced platelet aggregation in washed platelets at lower doses. Previous studies have reported that UCB at lower doses had no effect on platelet aggregation of platelet rich plasma, but it did inhibit at higher doses. This justifies that the albumin and some plasma proteins would bind UCB through non-covalent interaction, thus nullifying its effect on platelets. Further, the effect of UCB on platelet aggregation with collagen was confirmed with the platelet adhesion assay. In which, a significant inhibition UCB pre-treated collagen-coated wells and UCB pre-treated WP directly added to collagen-coated wells are observed. This might be due to the existence of an interaction between UCB and collagen. The results of the present study are contradictory to those presented in earlier studies that showed platelets undergoing activation upon exposure to increased levels of UCB in washed platelets^46^,^47^. This disparity in the results could be attributed to the difference in the protocols and solubility of UCB as discussed by Kundur *et al.*^48^.

Apoptotic platelets release negatively charged PS-positive plasma membrane vesicles, which are known as microparticles (MPs). MPs not only provide surface area for thrombus formation but also stimulate coagulation and influence vascular functions due to their pro-inflammatory nature. These events eventually lead to development of thrombotic disorders, CVDs and arthritis. Therefore, the future perspective of the study is to probe the link between hyperbilirubinemia and other pathophysiological conditions, especially inflammatory diseases. Overall the current study gives a better understanding of a novel approach to deal with thrombocytopenia associated with liver diseases with elevated UCB. Unconjugated hyperbilirubinemia should be taken more seriously and not treated as just one of the symptoms of liver diseases. Since UCB decreases platelet count by triggering platelet apoptosis via mitochondrial ROS-induced activation of p38 and p53, platelet protective antioxidants could be used to deal with thrombocytopenia in liver diseases. Moreover, the existing medications for liver diseases might themselves stimulate platelet apoptosis. Therefore, it is necessary to deal with hyperbilirubinemia and thrombocytopenia in human pathologies associated with the conditions of elevated unconjugated bilirubin.

## Materials and Methods

### Chemicals

All fluorogenic, non-fluorogenic dyes, Histopaque-1077 and standard platelet agonists were from Sigma Chemicals, USA. Monoclonal anti-cytochrome c antibody was from Epitomics, USA. z-DEVD-fmk, SB203580, ABT-737, Pifithrin-μ, antibodies against caspase-3, caspase-9, Bax, Bcl-2, BAD, phospho-BAD (ser136), Bid and tBid were from Santa Cruz Biotechnology, Inc., USA. Phospho-p38 (Thr180/Tyr182), p38, phospho-p53 (ser15), p53, COX-IV and β-tubulin antibodies were from Cell Signalling and Technology, USA. TNF-α, IL-1β and IL-6 antibodies were from Komabiotech, Korea. IL-10 and IL-23 antibodies were from Thermo Fisher Scientific, USA. LysoSensor green DND-189 was from Molecular probes, USA. Phenylhydrazine hydrochloride (PHZ) was from Fisher Scientific, USA. Commercial bilirubin and LDH estimation kits were purchased from Agappe Diagnostics Limited, India.

### Preparation of UCB solution

Unconjugated bilirubin (UCB, purity- 99%) used for the study was purchased from Sisco Research Laboratories, India. Purity of UCB was again confirmed by RP-HPLC which showed 99% pure (Data not shown). UCB was dissolved in dimethyl sulfoxide (DMSO)^48^, as UCB is partially soluble and forms precipitate in 0.02% cetyltrimethylammonium bromide (CTAB) and 0.1 N NaOH. Further, UCB solution was centrifuged at 4000 × *g* to pellet the precipitate and the concentration was determined by spectrophotometric method and using bilirubin estimation kit according to the manufacturer’s protocol. We found that there was no change in UCB concentration solubilised in DMSO, but there was decreased UCB concentration in CTAB and NaOH solubilised UCB. (Supplementary Figure 5). Therefore for the further assays, UCB was dissolved in DMSO to obtain a 10 mM stock solution from which the working solutions of 5 mM and 1 mM were prepared in DMSO. The final concentration of DMSO was limited to less than 4% in the reaction mixture. The UCB stock/working solutions were prepared in amber tubes and reactions were performed in dark/dim light to avoid photo oxidation of UCB. UCB solution was prepared freshly every time within 10 min, before treatment.

### Preparation of platelet-rich plasma and washed platelets

Venous blood was drawn from healthy volunteers (drug-free/non-smokers) with informed consent and was approved as per the guidelines of Institutional Human Ethical Committee (IHEC-UOM No. 40Res/2013-14), University of Mysore, Mysuru. It was immediately mixed with acid-citrate dextrose (ACD) anti-coagulant (2.5% tri-sodium citrate, 2% D-glucose, 1.5% citric acid) in the ratio of 7:1 (blood : ACD, v/v). The anti-coagulated blood was then centrifuged at 90 × *g* at 37 °C for 15 min and the supernatant thus obtained was the platelet-rich plasma (PRP). The PRP was centrifuged at 700 × *g* for 15 min at 37 °C. The platelet pellet thus obtained was suspended in CGS buffer (123 mM NaCl, 33 mM D-glucose, 13 mM tri-sodium citrate, pH 6.5) and washed thereafter at 700 × *g* for 15 min at 37 °C. The previous washing step was repeated one more time. Finally, the washed platelets (WP) were suspended in Tyrode’s buffer (2.5 mM HEPES, 150 mM NaCl, 2.5 mM KCl, 12 mM NaHCO_3_, 1 mM CaCl_2_, 1 mM MgCl_2_, 5.5 mM D-glucose, pH 7.4)^49^. The cell count was determined in both PRP and WP suspension using a Neubauer chamber and adjusted to 5 × 10^8^ cells/mL in the final suspension using platelet poor plasma/Tyrode’s buffer.

### Recruitment of hyperbilirubinemia (HB) subjects

HB subjects (n = 35) from Government Ayurvedic Medical College (GAMC), Mysuru were recruited for the study (Supplementary Table 1). HB subjects were recruited purely on basis of serum bilirubin level (reference range: 1.5–15 mg of total bilirubin/dL of blood). Cirrhotic, viral and obstructive jaundice patients were excluded from the study. All the experiments were in accordance and approved by the Institutional Human Ethical Committee (IHEC-UOM No. 47Res/2014-15), University of Mysore, Mysuru. Venous blood was drawn from HB subjects and non-smoking/drug-free healthy subjects (HS, n = 21) by trained professionals from GAMC, Mysuru. Platelets and serum samples were separated for further analysis. About ⅓ fraction of the drawn blood was used to separate serum and the remaining ⅔ fraction was immediately mixed with ACD in the ratio of 7:1 (blood: ACD, v/v). About 100 μL aliquots of anti-coagulated blood was used to determine cell count in an automated hemo-analyser (Sysmex KX-21, Japan). Further, the remaining anti-coagulated blood was then centrifuged to obtain PRP and WP. The cell count was determined in both PRP and WP suspension using a Neubauer chamber and adjusted to 5 × 10^8^ cells/mL in the final suspension using platelet poor plasma/Tyrode’s buffer. Serum was stored at −80 °C until further use.

### Phenylhydrazine (PHZ)-induced hyperbilirubinemia

Wistar rats of 4–6 week old were used for the experiments. The experimental animals were grouped as: Group I - Saline control; Group II - PHZ-induced, and each group consisted of 6 rats. Unconjugated hyperbilirubinemia was induced by injecting a single dose of PHZ (75 mg/kg) intraperitoneally and housed for two days^50^. Thereafter, animals were euthanized and blood was collected immediately through cardiac puncture. About ⅓ fraction of the collected blood was used to separate serum and the remaining ⅔ fraction was immediately mixed with ACD in the ratio of 5:1 (blood: ACD, v/v). About 100 μL aliquots of anti-coagulated blood was used to determine cell count in an automated hemo-analyser. Further, the remaining anti-coagulated blood was then centrifuged to obtain PRP and WP. The cell count was determined in both PRP and WP suspension using a Neubauer chamber and adjusted to 5 × 10^8^ cells/mL in the final suspension using platelet poor plasma/Tyrode’s buffer. Liver and spleen tissues from experimental animals were harvested and rinsed in ice cold saline, serum was separated and stored at −80 °C for further analysis.

### Flow cytometry

WP (1 × 10^6^ cells/mL) were independently treated with UCB (0–200 μM) and ABT-737 (1 μM) as positive control, and incubated for 30 min at 37 °C. After incubation, cells were stained using CM-H2DCFDA, JC-1, NAO (10-nonyl acridine orange) and Annexin V-FITC, washed and analysed using FACSVerse^TM^ flow cytometer (BD Biosciences, USA).

### Determination of reactive oxygen species (ROS)

Endogenous ROS generation in platelets was determined according to the method of Thushara *et al.*^51^. WP (5 × 10^6^ cells/mL) were taken separately in 96-well plates and treated with A23187 (1 μM) as positive control or UCB (0–200 μM) as test and the final volume was made up to 200 μL with Tyrode’s buffer and incubated for 30 min at 37 °C. For inhibition studies, pre-loaded platelets with UCB (200 μM), were incubated with Mito-TEMPO (10 μM) and incubated for 30 min at 37 °C. The untreated and treated platelets were then incubated with CM-H2DCFDA (10 μM) for 30 min at 37 °C and the fluorescence was recorded using a Varioskan multimode plate reader (Thermo Scientific, USA) by exciting the samples at 488 nm and measuring the resulting fluorescence at 530 nm. The same procedure was followed for untreated WP obtained from HS, HB and experimental animals.

### Estimation of intracellular calcium

Intracellular Ca^2+^ concentration was measured in WP according to the method of Thushara *et al.*^51^. WP (5 × 10^6^ cells/mL) were treated with A23187 (1 μM) or UCB (0–200 μM) and the final volume was made up to 200 μL with Tyrode’s buffer containing 1 mM Cacl_2_ and incubated for 30 min at 37 °C followed by incubation with fura-2/AM (2 μM). The fura-2/AM fluorescence was determined by exciting the samples at 340 and 380 nm and the resulting fluorescence was measured at 500 nm. Data were presented as absorption ratios (340/380 nm). The same procedure was followed for untreated WP obtained from HS, HB and experimental animals.

### Determination of changes in mitochondrial membrane potential (ΔΨm)

The cationic dye JC-1 was used to detect changes in the ΔΨ*m* according to the method of Thushara *et al.*^51^. WP (5 × 10^6^ cells/mL) were treated with UCB (0–200 μM) and the final volume was made up to 200 μL with Tyrode’s buffer and incubated for 30 min at 37 °C. The samples were loaded with JC-1 (2 μM) at 37 °C for 10 min. The cells were then excited at 488 nm and emission was detected at 585 nm for JC-1 aggregates and 516 nm for JC-1 monomers using multimode plate reader. Data were presented as emission ratios (585/516). The same procedure was followed for untreated WP obtained from HS, HB and experimental animals.

### Assessment of cardiolipin peroxidation

10-Nonyl acridine orange (NAO), a fluorescent probe was used to detect peroxidation of cardiolipin. NAO loses its affinity for peroxidised cardiolipin resulting in decreased fluorescence. WP (5 × 10^6^ cells/mL) were processed as discussed above with either A23187 (1 μM) or UCB (0–200 μM) and then loaded with NAO (1 μM) and incubated for 30 min at 37 °C. After incubation the fluorescence was recorded by exciting the samples at 499 nm and emission was recorded at 530 nm^52^. The same procedure was followed for untreated WP obtained from HS, HB and experimental animals.

### Assessment of mPTP formation

Formation of mPTP in platelets was assessed using calcein AM^53^. WP (5 × 10^6^ cells/mL) were treated with A23187 (1 μM), UCB (0–200 μM) and incubated for 30 min at 37 °C. Following incubation calcein AM (1 μM) along with CoCl_2_ (1 mM) for quenching cytosolic calcein fluorescence was added. Fluorescence was recorded by exciting the samples at 488 nm and emission was detected at 585 nm. The same procedure was followed for untreated WP obtained from HS and HB.

### Determination of PS externalization

PS externalization was determined according to the method of Thushara *et al.*^51^. WP (5 × 10^6^ cells/mL) were treated with UCB (0–200 μM) and for inhibition studies, pre-loaded platelets with UCB (200 μM) were incubated with Pifithrin-μ (5 μM)/z-DEVD-fmk (5 μM) and the final volume was made up to 200 μL with Tyrode’s buffer and incubated for 30 min at 37 °C. The untreated and treated platelets were stained with Annexin V-FITC (0.6 μg/mL) and fluorescence was measured in a multimode plate reader by exciting the samples at 496 nm and emission was recorded at 516 nm. The same procedure was followed for untreated WP obtained from HS, HB and experimental animals.

### Subcellular fractionation of platelets

UCB (0–200 μM) treated WP (1 × 10^8^ cells/mL) were suspended in isolation buffer (225 mM mannitol, 75 mM sucrose, 0.1 mM EGTA, 1 mg/mL fatty acid-free BSA, 10 mM HEPES-KOH, proteinase inhibitor mixture, phosphatase inhibitor mixture, pH 7.4) and were frozen in liquid nitrogen for 1 min and then thawed at 37 °C for 3 min. This freeze-and-thaw sequence was repeated for two more cycles, and then the samples were centrifuged at 700 × *g* for 10 min at 4 °C. The supernatant was further centrifuged at 15,000 × *g* for 10 min at 4 °C. The pellet was regarded as the mitochondria-rich fraction, and the supernatant was considered as the cytosolic fraction^54^.

### Immunobloting

To determine the expression level of various proteins, WP (1 × 10^8^ cells/mL) were treated with UCB (0–200 μM) and for inhibition studies, UCB (200 μM) treated platelets were pre-incubated with Mito-TEMPO (2 μM)/SB203580 (2 μM)/Pifithrin-μ (5 μM)/z-DEVD-fmk (5 μM)/ BSA (5 mg/mL) and incubated at 37 °C for 30 min. Further, platelet suspensions were lysed by adding 10 μL lysis buffer (20 mM Tris-HCl, pH 8, containing 0.5% Triton-X 100, 150 mM NaCl along with cocktail of protease inhibitors). Following centrifugation, the supernatants were separated on SDS-PAGE (4–14%) and proteins were transferred onto PVDF membrane. After blocking, the blots were probed with antibodies against phospho-p38 (Thy180/Tyr182), p38, phospho-p53 (ser15), p53, cyt. c, caspase-9, caspase-3, Bax, Bcl-2, BAD, phospho BAD, tBid, COX-IV, GAPDH and β-tubulin at 4 °C overnight. Blots were then incubated with horseradish-peroxidase (HRP)-conjugated secondary antibody and developed by enhanced chemiluminescence method and the bands were visualized using chemiluminescence imaging system (Alliance 2.7, Uvitec, UK). For re-probing, blots were incubated in stripping buffer (200 mM glycine, pH-2.2, 1% Tween-20 and 0.1% SDS) for 2 min and washed with TBST. Blots were again treated with stripping buffer for 5 min and washed thrice with TBST, 2 min each wash. The stripped membrane was blocked in TBST containing 5% non-fat milk powder over night at 4 °C and probed with desired antibodies^54^. The same procedure was followed for untreated WP obtained from HS, HB and experimental animals.

### Electron transport chain (ETC) assays

Assays for ETC complexes I, II, III and IV were performed as described previously^55^. Briefly, platelet mitochondria were isolated as described above and were treated with UCB (0–200 μM) along with vehicle control (DMSO). Complex I activity (NADH: ubiquinone oxidoreductase) was measured by the oxidation of NADH and the activity was expressed as mM NADH oxidised/min/mg protein. Complex II activity (succinate: ubiquinone oxidoreductase) was determined by the reduction of dichlorophenolindophenol (DCIP) and the activity was expressed as mM DCIP reduced/min/mg protein. Complex III activity (coenzyme Q: cytochrome c-oxidoreductase) was measured by cyt. c reduction and the activity was expressed as mM cyt. c reduced/min/mg protein. Complex IV (cyt. c oxidase) activity was measured by the oxidation of cyt. c and the activity was expressed as first-order rate constant (k) of mM cyt. c oxidized/min/mg protein.

### Evaluation of lysosomal acidity

For monitoring the changes in pH of lysosomes, pH sensitive dye LysoSensor Green DND-189 (LSG) was used^56^. LSG accumulates in intact lysosomes and its fluorescence decreases upon lysosome alkalinisation. WP (5 × 10^6^ cells/mL) were independently treated with UCB (0–200 μM) or A23187 (1 μM) and incubated for 30 min at 37 °C. Further untreated and treated samples were loaded with LSG (1 μM) and incubated for 30 min at 37 °C. Fluorescence was recorded by exciting the samples at 488 nm and emission at 510 nm. The same procedure was followed for untreated WP obtained from HS and HB.

### Estimation of glucose-6-phosphate dehydrogenase (G6PDH) enzyme activity

G6PDH activity was determined in WP treated with either A23187 (1 μM) or UCB (0–200 μM) and incubated for 30 min at 37 °C. After incubation, platelets were pelleted, suspended in distilled water and lysed by sonication. Activity was monitored by adding platelet lysate (50 μg) to 1 mL reaction volume (50 mM Tris-HCl, pH 7.5 containing 3.8 mM NADP, 3.3 mM glucose-6-phosphate and 6.3 mM MgCl_2_). Increase in absorbance was recorded at 340 nm for 3 min due to NADP^+^-dependent glucose 6-phosphate transformation and the activity was expressed as mM NADPH formed/min/mg protein^57^.

### Measurement of γ-Glutamyltransferase (GGT) activity

To determine the GGT activity, WP (5 × 10^6^ cells/mL) were treated with either A23187 (1 μM) or UCB (0–200 μM) and incubated for 30 min at 37 °C. After incubation, platelets were pelleted, suspended in distilled water and lysed by sonication. The resulting lysate was used to determine GGT activity according to the method of Sener *et al.*^16^. Activity was monitored in 1 mL reaction volume consisting of γ-glutamyl-*p*-nitroanilide (4 mM), glycylglycine (40 mM) in Tris-HCl buffer (185 mM, pH 8.2). The results were calculated using molar extinction coefficient for *p*-nitroanilide (9,900 M^−1^cm^−1^) at 405 nm expressed as mM *p*-nitroanilide formed/min/mg protein. The same procedure was followed for untreated WP obtained from HS and HB.

### Measurement of LDH leakage

WP (5 × 10^6^ cells/mL) were treated either with UCB (0–200 μM) or with A23187 (1μM) for 30 min at 37 °C and platelets were pelleted by centrifugation at 3,700 × *g* for 10 min. Supernatants were used to detect LDH release by using LDH kit, according to the manufacturer’s protocol. The assay was performed in a time course of decrease in NADH absorbance at 340 nm for 3 min.

### Evaluation of platelets viability by MTT assay

MTT colorimetric assay was performed to assess the cell viability^58^. WP (1 × 10^6^ cells/mL) was taken separately in 96-well microtiter plates and treated with either A23187 (1 μM) or with UCB (0–200 μM) and the final volume was made up to 200 μL with Tyrode’s buffer. After 30 min of incubation, 250 μM of MTT was added and incubated for additional 3 h. Thereafter, MTT was removed and remaining formazan crystals were completely dissolved in DMSO and the absorbance was recorded at 570 nm.

### Platelet aggregation assay

Platelet aggregation was determined by turbidimetric method with a dual channel Chrono-log model 700-2 aggregometer (Havertown, USA). Briefly, 250 μL of WP (1.5 × 10^8^ cells/mL) was taken in siliconized glass cuvette and pre-incubated for 5 min at 37 °C with UCB (0–200 μM), and the aggregation was initiated by the addition of collagen (2 μg/mL). The aggregation was then followed with constant stirring at 1200 rpm for 6 min at 37 °C^59^.

### Platelet adhesion assay

Platelet adhesion assay was performed according to the method by Kumar *et al.*^60^. Briefly, 20 μg of collagen type I in 200 μL PBS was added independently to 96-well polystyrene microtiter plates and kept for 16 h at 4 °C. The coated wells were then blocked by adding 200 μL of 1% BSA in PBS for 1 h at 37 °C and washed with PBS. In the first set of experiment, UCB (0–200 μM) was directly added to the collagen type I coated wells, incubated for 10 min followed by washing with PBS and then WP (1.5 × 10^8^ cells/mL) was added. In the second set of experiment, WP pre-treated with UCB (0–200 μM) for 10 min at 37 °C was added to the pre-coated collagen type I wells. The total reaction volume was made up to 200 μL with PBS. The reaction mixture was incubated at 37 °C for 90 min and then washed with PBS. The adherent platelets were then lysed by adding 150 μL lysis buffer (100 mM citrate buffer pH 5.4 containing 5 mM *p*-nitrophenyl phosphate and 0.1% Triton X-100) at 37 °C for 90 min. The reaction was terminated by inactivating the membrane bound acid phosphatase with the addition of 100 μL stopping reagent (2 N NaOH). The colour developed was measured at 405 nm. Platelet adhesion was expressed as percent adhesion, considering PBS-treated platelet suspension as 100%.

### Macrophage engulfment assay

Engulfment of apoptotic platelets from monocyte derived macrophage was assessed as described by Kumari *et al.*^61^. In brief, blood drawn from healthy donors (drug-free/non-smokers) was collected in citrate containing tube, and peripheral blood mononuclear cells (PBMCs) were isolated using Histopaque-1077 according to the standard protocol from the manufacturer. The obtained PBMCs were plated onto polystyrene culture flasks for 4 h at 37 °C and washed with PBS to remove non-adherent lymphocytes. Monocytes (200,000 in 500 μL volume) were then plated on to 6-well plates in RPMI 1640 medium supplemented with 10% fetal bovine serum and cultured for 7 days to obtain monocyte-derived macrophages. Control and UCB (200 μM) - treated platelets labelled with calcein-AM were incubated with adherent monolayer of monocyte-derived macrophages for 45 min. Following the incubation period, the phagocyte monolayer was washed to remove non-interacting platelets, and adherent macrophages were removed by treatment with trypsin at 37 °C for 5 min, followed by 5 mM EDTA at 4 °C. Monocyte-derived macrophages were recovered by trypsin-EDTA treatment for 15 min at 37 °C and subjected to flow cytometric analysis.

### Histological assessment of liver and spleen tissues

The liver and spleen tissues were dissected out and blotted free of blood, rinsed in ice-cold saline and fixed in 10% buffered formalin for overnight. The tissue samples were subjected to dehydration by processing with different grades of alcohol and chloroform mixture. The processed tissues were embedded in paraffin wax, and sections (5 μm thickness) were prepared, stained with hematoxylin- eosin dye (H&E) and observed under an Axio imager.A2 microscope (Oberkochen, Germany) and photographed.

### Protein estimation

The protein estimation was done according to the method of Lowry *et al.*^62^ using BSA as standard.

### Statistical analysis:

All the results were expressed as mean ± SEM of five independent experiments. Statistical significance among groups was determined by t-test and one way analysis of variance (ANOVA) followed by Tukey’s test for comparison of means as appropriate.

## Additional Information

**How to cite this article**: NaveenKumar, S. K. *et al.* Unconjugated Bilirubin exerts Pro-Apoptotic Effect on Platelets *via* p38-MAPK activation. *Sci. Rep.* **5**, 15045; doi: 10.1038/srep15045 (2015).

## Supplementary Material

Supplementary Information
