# Supplementary material for: Unconjugated Bilirubin exerts Pro-Apoptotic Effect on Platelets via p38-MAPK activation
Source: Sci Rep. 2015 Oct 13;5:15045. doi: 10.1038/srep15045 (PMC4602209; doi:10.1038/srep15045)
Supplement: Supplementary Information [file srep15045-s1.pdf]

Unconjugated Bilirubin exerts Pro-Apoptotic Effect on Platelets *via* p38-MAPK activation

Somanathapura K. NaveenKumar[1], Ram M. Thushara[1], Mahalingam S. Sundaram[1], Mahadevappa Hemshekhar[1,2], Manoj Paul[1], Chinnasamy Thirunavukkarasu[3], Basappa[4], Ganesh Nagaraju[5], Sathees C. Raghavan[5], Kesturu S. Girish[1,6]*, Kempaiah Kemparaju[1]*, Kanchugarakoppal S. Rangappa[7]*

[1]DOS in Biochemistry, University of Mysore, Manasagangothri, Mysuru-570 006, India; [2] Department of Internal Medicine, Manitoba Centre for Proteomics and Systems Biology, University of Manitoba, Winnipeg-R3E3P4, Canada; [3]Department of Biochemistry and Molecular Biology, Pondicherry Central University, Pondicherry-605 014, India; [4]Laboratory of Chemical Biology, Department of Chemistry, Bangalore University, Bengaluru-560 056, India; [5]Department of Biochemistry, Indian Institute of Science, Bengaluru-560 012, India; [6]Department of Studies and Research in Biochemistry, Tumkur University, Tumkur-572 103, India; [7]DOS in Chemistry, University of Mysore, Manasagangothri, Mysuru-570 006, India;

***Corresponding authors**

Address of correspondence

1) **Dr. Kanchugarakoppal S. Rangappa**
   Professor
   DOS in Chemistry, University of Mysore, Karnataka, INDIA
   E-mail: rangappaks@gmail.com Tel.: +91-821-2419666
2) **Dr. Kempaiah Kemparaju**
   Professor
   DOS in Biochemistry, University of Mysore, Karnataka, INDIA
   E-mail: kemparajuom@gmail.com Tel.: +91-9945996543
3) **Dr. Kesturu S. Girish**
   Associate Professor
   Department of Studies and Research in Biochemistry
   Tumkur University, Tumkur-572 103, Karnataka INDIA
   University of Mysore, Mysuru-570 006, Karnataka, INDIA
   E-mail: ksgbaboo@gmail.com Tel.: +91-9964080540

Supplementary information

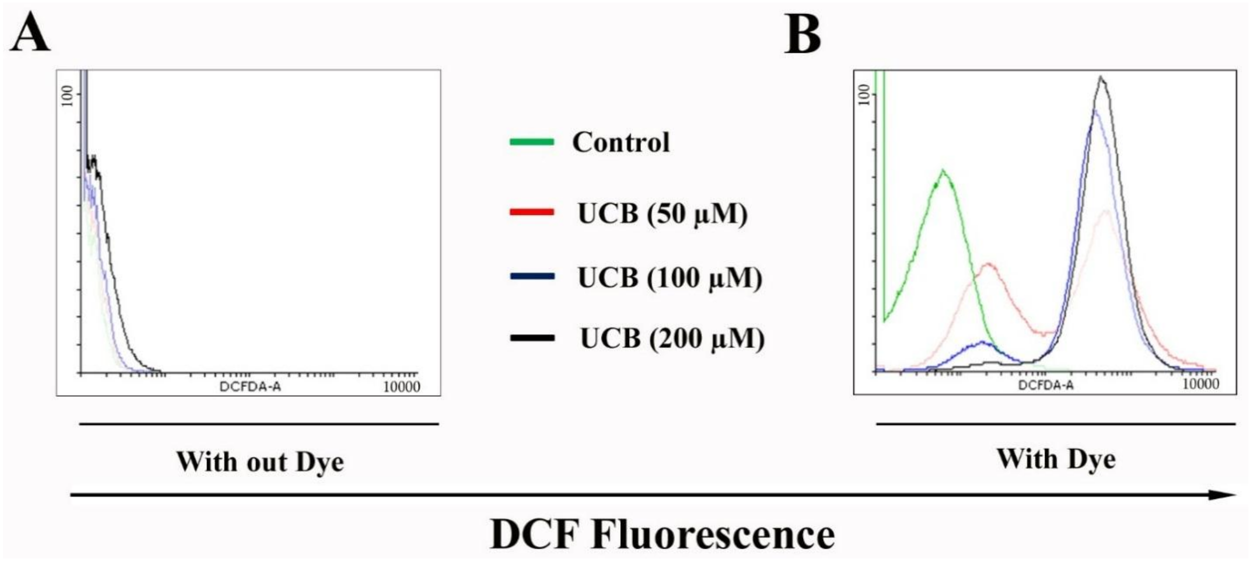

Supplementary Figure 1: FACS analysis of UCB treated platelets in presence/absence of DCFDA. Flow cytometric analysis of UCB treated platelets (**A**) in absence of DCFDA and (**B**) in presence of DCFDA.

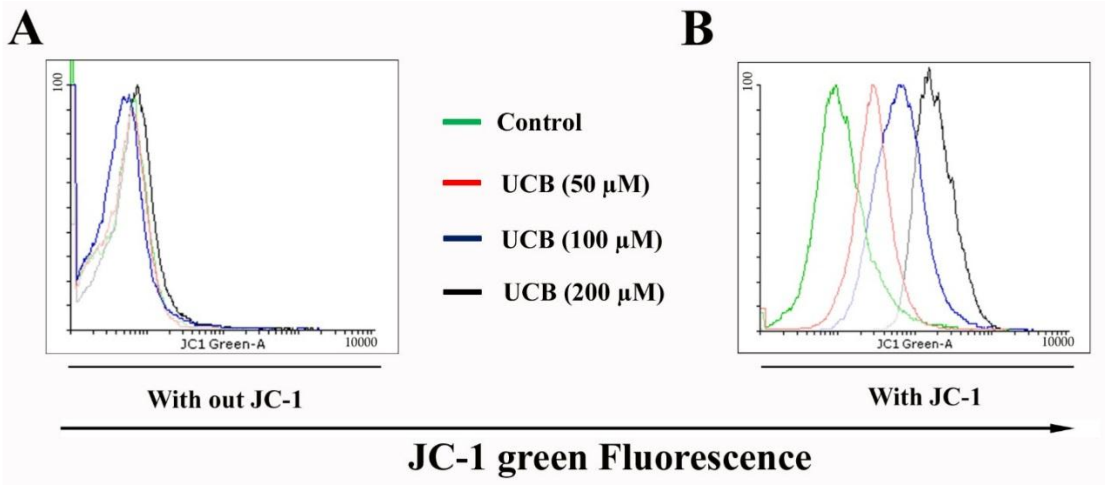

Supplementary Figure 2: FACS analysis of UCB treated platelets in presence/absence of JC-1. Flow cytometric analysis of UCB treated platelets (**A**) in absence of JC-1 and (**B**) in presence of JC-1.

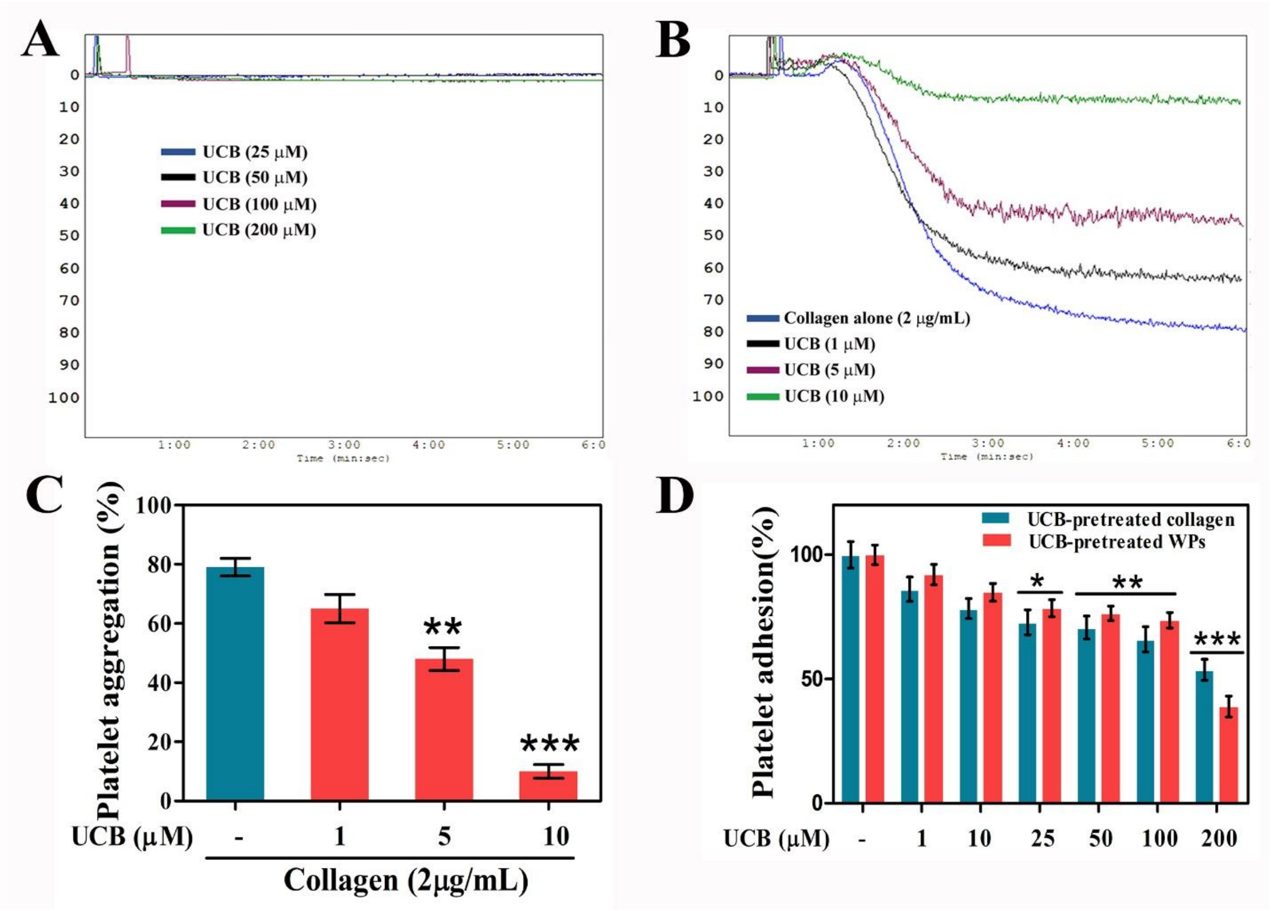

Supplementary Figure 3: Effect of UCB on platelet aggregation and adhesion. Effect of UCB on platelet aggregation induced by **(A)** UCB alone and **(B)** Collagen. **(C)** Graphical representation of percentage platelet aggregation with collagen (2μg/mL) in presence or absence of UCB. **(D)** Effect of UCB on platelet adhesion on immobilized collagen type I. *p**< 0.05, *p***< 0.01, *p****< 0.001; significant compared to control platelets.

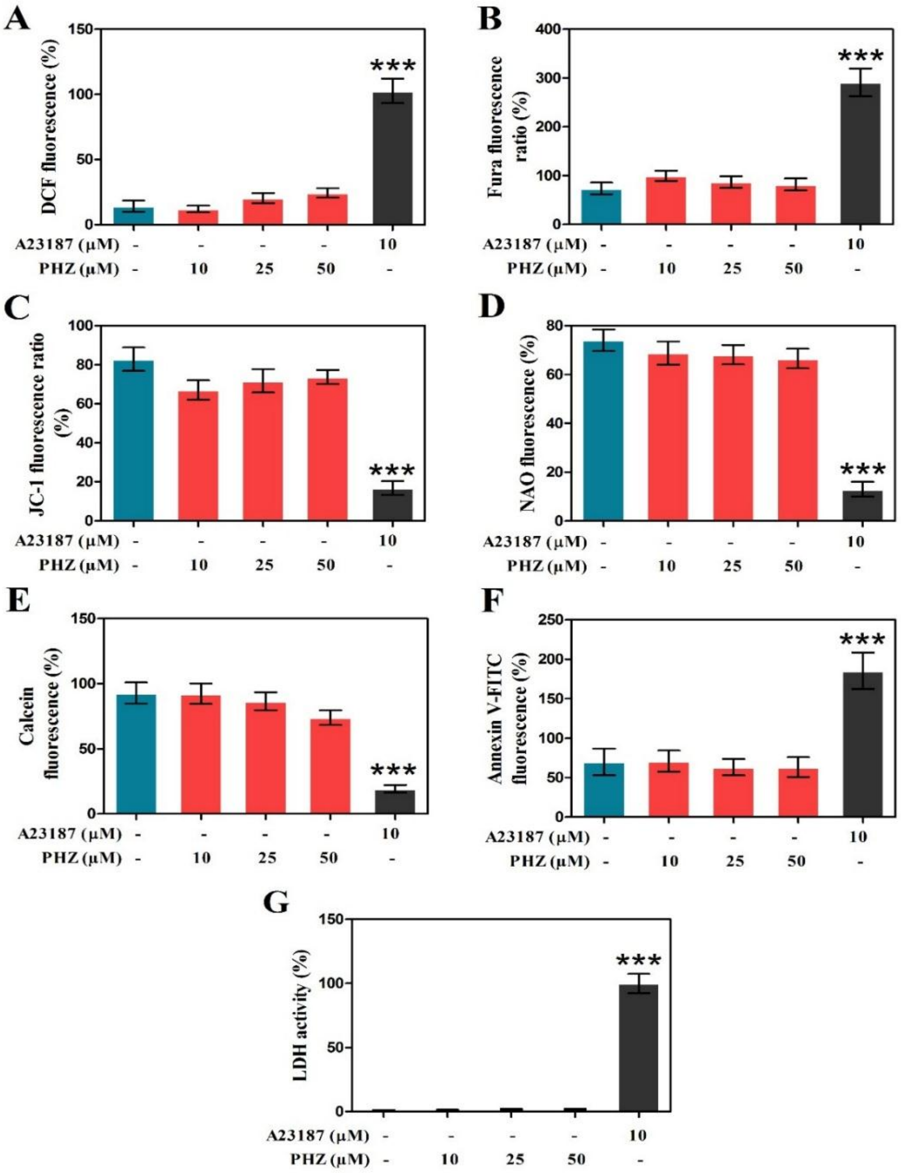

Supplementary Figure 4: Effect of PHZ on platelet apoptotic markers *in vitro*. Platelets were treated with the dose that corresponds to the concentration of PHZ in circulation during *in vivo* administration of PHZ and the following markers were determined. **(A)** ROS, **(B)** intracellular calcium levels, **(C)** mitochondrial membrane potential, **(D)** cardiolipin peroxidation, **(E)** mPTP formation and **(F)** PS externalization. Values are presented as mean ± SEM (n=5), and expressed as percentage increase/decrease in fluorescence. **(G)** LDH activity. *p****< 0.001; significant compared to A23187 treated platelets.

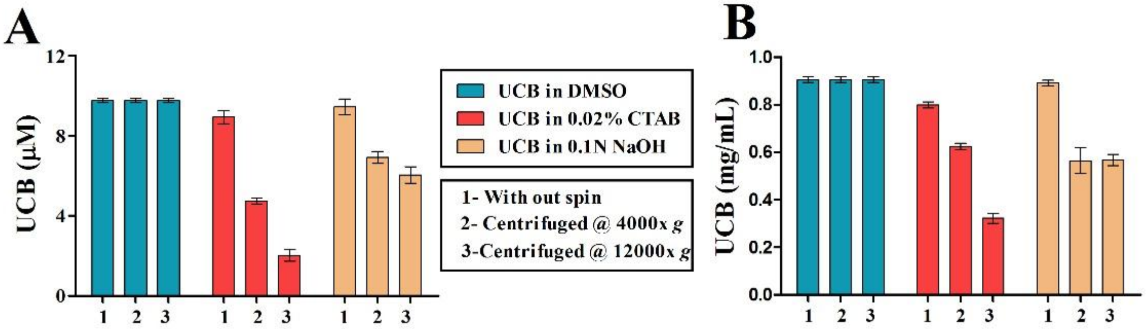

Supplementary Figure 5: Determination of the concentration of bilirubin. The concentration of bilirubin dissolved in different solvents was estimated by **(A)** spectrophotometry and **(B)** bilirubin estimation kit.

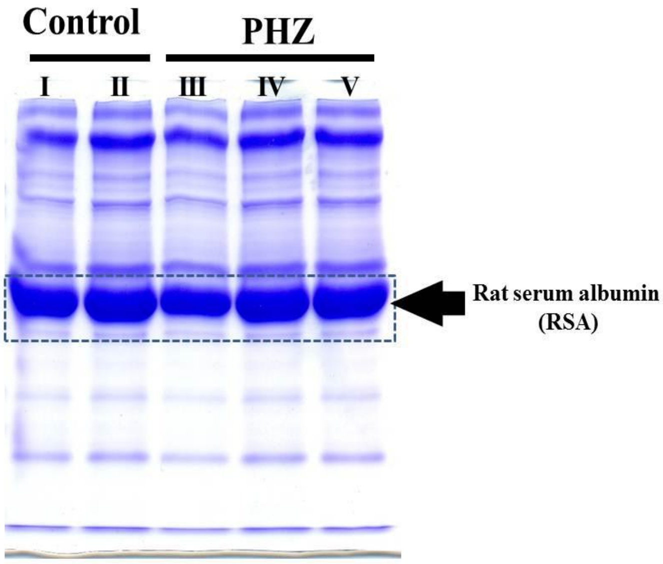

Supplementary Figure 6: Full length image of SDS-PAGE from figure 7. Regions of interest are highlighted and are presented as cropped image in figure 7. Samples are as follows; Lane I & II- control group serum, Lane III, IV & V- PHZ treated group serum.

Supplementary Table 1. Clinical characteristics of HS and HB patients.

Characteristics		HS	HB
	**Total subjects**	n = 21	n = 35
	**Gender (Males/Females)**	16/05	29/06
**PLT count ($x10^3/\mu L$)**	Total subjects	327.61 ± 39.37	148.14 ± 52.65
	Male	330.56 ± 42.30	142.62 ± 55.15
	Female	318.20 ± 25.84	172.50 ± 24.85
**UCB concentration (mg/dL)**	Total subjects	0.40 ± 0.21	1.57 ± 1.41
	Male	0.42 ± 0.22	1.62 ± 1.19
	Female	0.33 ± 0.13	2.17 ± 1.91

Information in the above table were represented as follows: Data presented for Platelets as mean count ± SD; UCB as mean concentration in mg/dL ± SD
